# Using High-Content Screening to Generate Single-Cell Gene-Corrected Patient-Derived iPS Clones Reveals Excess Alpha-Synuclein with Familial Parkinson’s Disease Point Mutation A30P

**DOI:** 10.3390/cells9092065

**Published:** 2020-09-10

**Authors:** Peter Barbuti, Paul Antony, Bruno Santos, François Massart, Gérald Cruciani, Claire Dording, Jonathan Arias, Jens Schwamborn, Rejko Krüger

**Affiliations:** 1 Translational Neuroscience, Luxembourg Centre for Systems Biomedicine, University of Luxembourg, L-4362 Luxembourg, Luxembourg; paul.antony@uni.lu (P.A.); Bruno.Santos@uni.lu (B.S.); francois.massart@uni.lu (F.M.); gerald.cruciani@uni.lu (G.C.); claire.dording@lih.lu (C.D.); 2 Transversal Translational Medicine, Luxembourg Institute of Health, L-1445 Luxembourg, Luxembourg; 3 Department of Pathology and Cell Biology, Columbia University Irving Medical Center, New York, NY 10032, USA; 4 Developmental and Cellular Biology, Luxembourg Centre for Systems Biomedicine, University of Luxembourg, L-4362 Luxembourg, Luxembourg; arias@stem.lu (J.A.); jens.schwamborn@uni.lu (J.S.); 5 Department of Biosciences and Nutrition, Neo, Karolinska Institutet, SE-141 83 Huddinge, Sweden; 6 Department of Neurodegenerative diseases, Hertie Institute for Clinical Brain Research, University Clinics Tübingen, 72076 Tübingen, Germany; 7 Parkinson Research Clinic, Centre Hospitalier de Luxembourg (CHL), L-1210 Luxembourg, Luxembourg

**Keywords:** CRISPR-Cas9, high-content screening (HCS), fluorescent-activated cell sorting (FACS), Parkinson’s disease (PD), patient-derived iPS, single-cell clones, isogenic cell lines, *SNCA*, alpha-synuclein, A30P

## Abstract

The generation of isogenic induced pluripotent stem cell (iPSC) lines using CRISPR-Cas9 technology is a technically challenging, time-consuming process with variable efficiency. Here we use fluorescence-activated cell sorting (FACS) to sort biallelic CRISPR-Cas9 edited single-cell iPSC clones into high-throughput 96-well microtiter plates. We used high-content screening (HCS) technology and generated an in-house developed algorithm to select the correctly edited isogenic clones for continued expansion and validation. In our model we have gene-corrected the iPSCs of a Parkinson’s disease (PD) patient carrying the autosomal dominantly inherited heterozygous c.88G>C mutation in the *SNCA* gene, which leads to the pathogenic p.A30P form of the alpha-synuclein protein. Undertaking a PCR restriction-digest mediated clonal selection strategy prior to sequencing, we were able to post-sort validate each isogenic clone using a quadruple screening strategy prior to generating footprint-free isogenic iPSC lines, retaining a normal molecular karyotype, pluripotency and three germ-layer differentiation potential. Directed differentiation into midbrain dopaminergic neurons revealed that *SNCA* expression is reduced in the gene-corrected clones, which was validated by a reduction at the alpha-synuclein protein level. The generation of single-cell isogenic clones facilitates new insights in the role of alpha-synuclein in PD and furthermore is applicable across patient-derived disease models.

## 1. Introduction

Parkinson’s disease (PD) is the most common neurodegenerative movement disorder. The global burden of PD, assessed in 2016 was 6.1 million patients, this is estimated to reach 12 million patients worldwide by 2050 [1]. PD is clinically characterized by the two neuropathological features, the degeneration of innervating A9 dopaminergic neurons from the substantia nigra pars compacta (SNc) to the striatum in the midbrain, and the formation of intracytoplasmic neuronal inclusion bodies, referred to as Lewy bodies that are immunopositive for the alpha-synuclein protein in the neurons that remain [2].

PD has a heritability of between 10 and 15% with mutations in several genes explaining between 5 and 10% of these familial cases. The first PD gene identified was *SNCA* that encodes alpha-synuclein identified as an autosomal dominant form of the disease. Rare and highly penetrant missense mutations in the *SNCA* protein at p.A53T [3], p.A30P [4], p.E46K [5], p.G51D [6] and p.A53E [7] have all been identified. Similarly, duplications [8,9] and triplications [10] of the *SNCA* gene locus are pathogenic, with the triplication having more severe clinical symptoms and faster disease progression than the duplication [11]. In sporadic PD, a 4-fold increase in alpha-synuclein mRNA has been found in the SNc in PD patients at post-mortem in comparison to unaffected controls [12]. Together, this indicates that increased levels of wild-type alpha-synuclein may be sufficient to cause disease. Additionally, multiple genome wide association studies (GWAS) have identified common single nucleotide polymorphism (SNP) genetic variants in *SNCA* as a risk factor in sporadic PD related to the modulation of alpha-synuclein expression [13,14,15].

The advancement in gene-editing by utilizing the RNA guided Cas9 nuclease from the clustered regularly interspaced short palindromic repeats (CRISPR) of the bacterial adaptive immune system in human cells has revolutionized disease modeling [16,17]. Genome editing by CRISPR-Cas9 generates a double-strand break (DSB) in which the error-free homology-directed repair (HDR) as opposed to the error-prone non-homologous end-joining (NHEJ) is used to repair the DNA. Consequently, this allows the gene editing and correction of pathological missense mutations to take place in-vitro, thereby isolate, and determine the exact effect of the specific mutation in relation to its isogenic corrected control.

The generation of gene-corrected patient-derived isogenic induced pluripotent stem cell (iPSC) lines in recent years typically involves strategies successfully using antibiotic resistance combined with fluorescence activated cell sorting (FACS) technology, before embarking on a screening and sequencing campaign to select, if successful, the isogenic clone [18,19]. Recently, the biallelic genomic editing technique has successfully used FACS in addition to antibiotic resistance to both introduce and gene-correct heterozygous mutations [20,21,22]. CRISPR-Cas9 gene knockout at the genomic scale has been achieved in cell line models [23] and patient-derived iPSC models [24], however the interlinked generation of footprint-free loci-specific gene modifications with single cell cloning in a high throughput manner remains so far challenging.

Due to the technical complexity of gene-editing cultured human iPSCs, isogenic cell lines are generated as a polyclonal cell population, an advantage here is that higher cell numbers are achieved earlier in the derivation process. In contrast, a disadvantage of polyclonal isogenic cell lines is that the cells within the colony can have different proliferation rates. This is of particular importance where the gene editing concerns a developmental, cell-cycle or cell-death affected mutation, which over the course of the culture and repeated passaging, changing cellular composition can take place leading to variance in the research findings. Characterized single-cell clones offer the certainty of having a healthy genetic background, absent of biases in different proliferation rates. The quality control provided by the generation of single-cell isogenics ensures the reliability of phenotyping assays necessary for future drug discovery and translational research.

In this study, we gene-corrected the PD patient-derived iPS cells containing the heterozygous c.88G>C mutation in *SNCA* that generates the pathogenic A30P alpha-synuclein protein. We apply the biallelic genomic editing technique of antibiotic resistance selection followed by triple-reporter FACS technology to sort the CRISPR-Cas9 gene-edited iPSCs with a single-cell iPS clone per well of a 96-well plate. We then developed an algorithm and used OPERA^®^ (PerkinElmer Inc., Waltham, MA, USA) high-content screening (HCS) technology to post-sort screen the correctly edited clones prior to cell culture expansion. For clonal selection, we exploited that the c.88G>C p.A30P *SNCA* mutation itself leads to the creation of an *Mva1* restriction digest site within the exon 2 of the *SNCA* gene [4], thereby negating the need to generate additional silent mutations that would be required for the PCR-mediated clonal selection process. Thereby using PCR amplification and restriction digestion, we use the undigested PCR product as a way to validate the gene-corrected clones. Using this validation process of HCS and restriction digestion, we are able to quickly and cost-effectively generate and screen our single-cell iPSC clones (12 of the picked and amplified 34 clones were correctly edited, efficiency rate = 35.30%). We then performed Sanger sequencing as a validation step to confirm the gene editing, before excising the transcript and genotyping the footprint-free isogenic iPSC lines—a gene editing process that can be completed within 90 days post-transfection. We fully characterized two randomly selected iPSC clones to ensure that the cells retained pluripotency, and furthermore directly differentiated these iPSCs, together with the founder line containing the p.A30P *SNCA* mutation into ventral midbrain dopaminergic neurons (vmDANs) via a neural precursor cell (NPC) stage in order to generate the functional alpha-synuclein protein.

We show that the generation of single-cell isogenic human iPSC lines assisted by the quadruple selection strategy (QSS) validation of: antibiotic resistance, single-cell FACS, PCR amplification and restriction-digestion mediated clonal selection, and finally Sanger sequencing is a process that facilitates the real-time tracking of the edited clones. This process not only increases the traceability of the de-novo single-cell isogenic cell line but increases the efficiency compared to previous methods [18,25]. Furthermore allowing the operator to track the successfully edited clone(s) through each stage of the QSS process aids accuracy, speed and efficiency of the isogenic iPSC generation, a current limitation within the genome-editing field, a strategy applicable across multiple research areas within disease modeling.

## 2. Materials and Methods

### 2.1. Cell Line and Ethical Approval

Skin biopsies were obtained after informed consent from a patient with Parkinson’s disease. The patient carried a heterozygous mutation c.88G>C in the *SNCA* gene generating the pathogenic p.A30P form of the alpha-synuclein protein. This patient was an affected sibling of the index patient from the 1998 study [4,26]. The generation and characterization of induced pluripotent stem cells (iPSCs) from the dermal fibroblasts has been described [27] and has a unique identifier HIHDNDi001-B (https://hpscreg.eu/cell-line/HIHDNDi001-B). Ethical approval for the development of and research pertaining to patient-derived cell lines have been given by the National Committee for Ethics in Research, Luxembourg (Comité National d’Ethique dans la Recherche; CNER #201411/05).

### 2.2. Maintenance of iPSCs

iPSCs were routinely cultured in 6-well plates (Nunc, Roskilde, Denmark; 140675). These were coated with high concentration growth factor reduced Matrigel^®^ (1:100; Corning, New York, NY, USA; 354263) according to the manufacturer instructions. The iPSCs were maintained in E8 media, as previously described [27,28] (DMEM/F12 + HEPES; Life Technologies, Thermo Fisher Scientific, Waltham, MA, USA; 31330038), Insulin-Transferrin-Selenium (ITS; 1%; Life Technologies, Thermo Fisher Scientific, Waltham, MA, USA; 41400045), Penicillin-Streptomycin (1%; Life Technologies, Thermo Fisher Scientific, Waltham, MA, USA; 15140), L-Ascorbic acid 2-phosphate sesquimagnesium salt hydrate ((AA2PM); 64 µg/mL; Sigma-Aldrich, St. Louis, MO, USA; A8960), bFGF (10 ng/mL; Peprotech, Rocky Hill, NY, USA; 100-18B), TGF-β1 (2 ng/mL; Peprotech, Rocky Hill, NJ, USA; 100-21) and Heparin (100 ng/mL; Sigma-Aldrich, St. Louis, MO, USA; H3149). The iPSCs were maintained as colonies and passaged in Dispase^®^ (5 U/mL; CellSystems GmbH, Troisdorf, Germany; LS02104).

### 2.3. Generation of Isogenic Cell Lines—Insertion of Fluorescent Constructs

**Figure 1:**
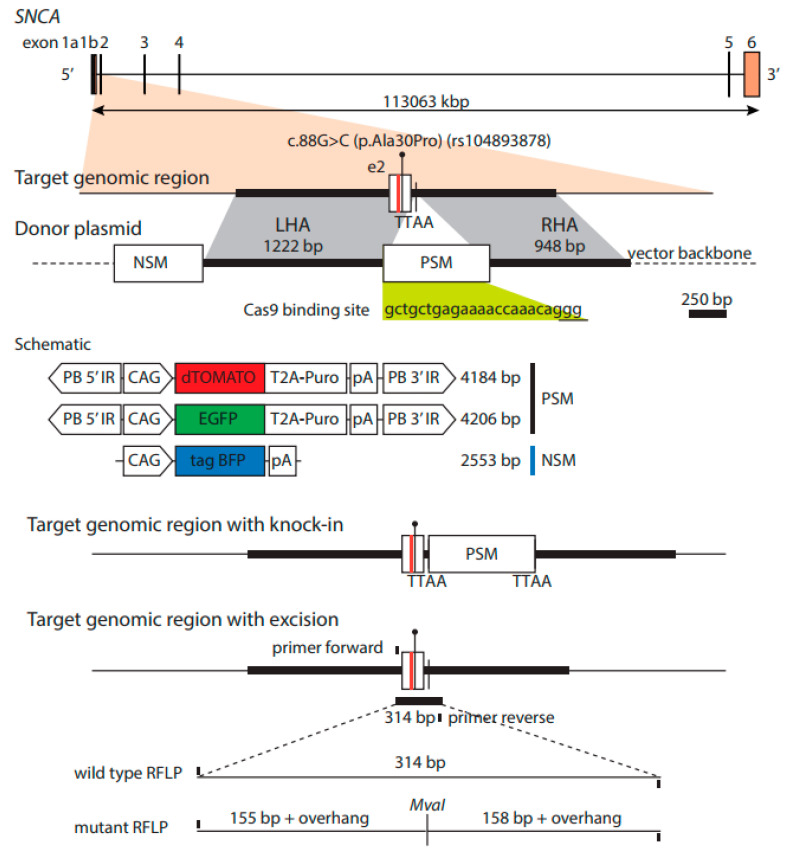
Donor vector for homology directed repair. The c.88G>C mutation is in the target genomic region of Exon 2 of the SNCA gene located on chromosome 4. The vector backbone of the two constructs contain the tagBFP outside the homology arms, within the homology arms is the wildtype genomic DNA with the dTOMATO or EGFP fluorescent constructs.

To generate isogenic stem cells through biallelic targeting, two plasmids were used. Both plasmids target a 2170 bp homology sequence of the *SNCA* gene, split to a left homology arm of 1222 bp and a right homology arm of 948 bp. The targeted genomic sequence is free of complex repetitive elements. The homology arms code the wild type *SNCA* gene sequence and flank the positive selection module structure of plasmids Addgene 100,603 and 100,604. This results in a donor plasmid expressing dTOMATO-TA-Puromycin, and another expressing EGFP-TA-Puromycin (Addgene, 85845). The donor plasmids were knock-in using the well-established Cas9 plasmids pX330 (Addgene, 42230) containing an sgRNA targeting the human *SNCA* gene sequence gctgctgagaaaaccaaaca, shown in Figure 1. For the knock-in, iPSCs were dissociated to single cells using Accutase and plated in iPS media plus Rho-Kinase Inhibitor Y-27632 (10 μM; Abcam, Cambridge, UK; Ab120129). Then, 1 × 10^6^ iPSCs were electroporated once using the 2D-Amaxa nucleofector unit (Lonza, Basel, Switzerland) with program B16. A mass of 1.5 μg of each donor plasmid and 2.5 μg of Cas9 plasmid were used together for the electroporation. The Human Stem Cell Nucleofector Kit 1 (Lonza, Basel, Switzerland) was used for the transfection according to the manufacturer’s instructions. After electroporation, 1 mL of E8 was added to the cuvette before being placed in the incubator for 10 min. The cells were then plated into three wells of a 6-well plate with approximately 1.5 mL of media per well. Cell selection using antibiotic resistance to puromycin (Sigma-Aldrich, St. Louis, MO, USA; P9620) was used as soon as small to medium-sized colonies began to appear. Puromycin concentrations of 0 μg/mL, 0.5 μg/mL and 1 μg/mL were used respectively in the three plated wells for 24 h with the viable colonies following 1 μg/mL antibiotic treatment passaged and then expanded into 2 × 10 cm^2^ tissue culture treated Matrigel™-coated dishes (Nunc, Roskilde, Denmark; Z755923) prior to sorting.

### 2.4. Generation of Isogenic Cell Lines—Sorting Single-Cell iPSCs

iPSC colonies were dissociated to single cells using Accutase before being centrifuged (300× *g*; 3 min) and resuspended in sterile-filtered sorting buffer (PBS containing EDTA (1 mM; Sigma-Aldrich, St. Louis, MO, USA; E9884), HEPES (20 mM, Life Technologies, Thermo Fisher Scientific, Waltham, MA, USA; 15630), bovine serum albumin (0.2%, Sigma-Aldrich, St. Louis, MO, USA; A2058), 1% Penicillin/Streptomycin and 10 μM of the Rho-Kinase inhibitor. Cells were sorted at 4 °C using an 85 μM nozzle and a neutral density filter of 1.5 on the BD FACS Aria™ III (BD Biosciences, Franklin Lakes, NY, USA). Cytometer Setup and Tracking (CST) beads were used daily to calibrate and define the baseline performance of the machine using the FACSDiva™ software. Prior to sorting, the drop delay experiment was setup to calibrate the sorting efficiency using the Accudrop™ beads; this was manually adjusted until the efficiency was ≥ 99.5%. In preparation of the single-cell sorting in a 96-well plate (Nunc, Roskilde, Denmark, delta surface treated #167008) a calibration plate was placed onto the cooled stage to setup the sorting parameters. Briefly, the single-cell mode was selected in the FACSDiva™ software and 100 beads were sorted per well in order to visually check if the cells were sorted to the centre of the well, or if further calibration was required. Once the visual inspection was passed, a 96-well plate precoated with Matrigel, containing 100 μL of E8 iPSC media and the Rho-Kinase inhibitor was placed onto the stage with 1 cell/well selected in the experimental setup parameters. The cells once sorted were left in the incubator (37 °C, 5% CO_2_) for 48 h before the media being half-changed daily; the Rho-Kinase inhibitor was not included in the media at this stage. The cells were maintained for 10–21 days before they appeared visible and were able to be screened on the OPERA^®^ High-Content Screening (HCS) System (PerkinElmer Inc., Waltham, MA, USA).

**Figure 2:**
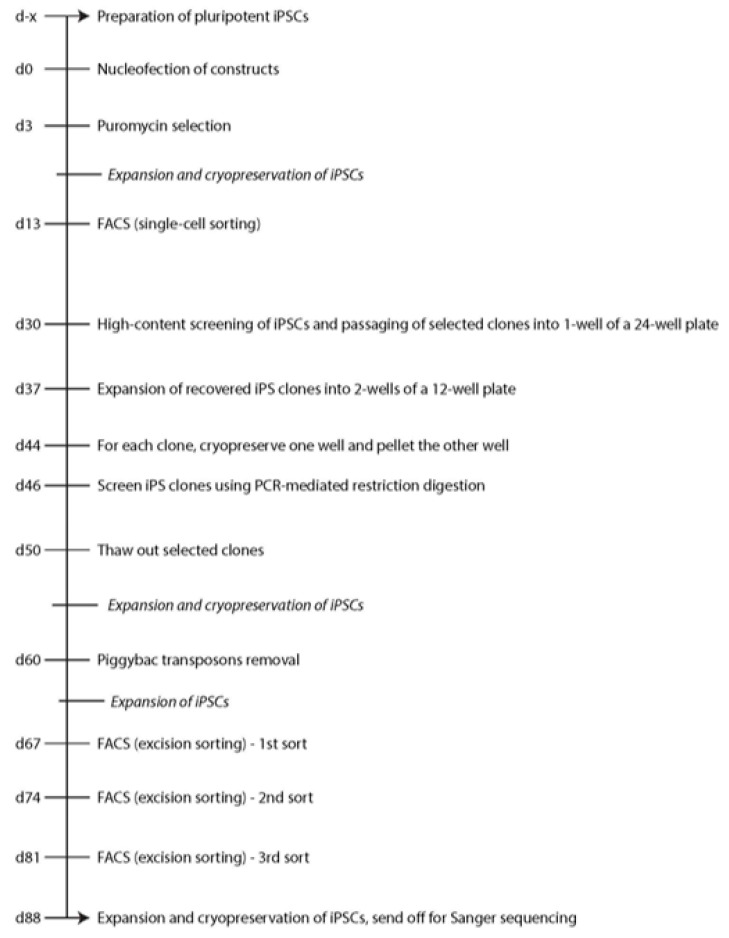
Timeline for the generation of single-cell gene-corrected isogenic iPS cell lines.

A timeline for the generation of the single-cell isogenic stem cell lines is shown in Figure 2.

### 2.5. Generation of Isogenic Cell Lines—Expansion of the Single-Cell Clones

Visual inspection was used to select the clones for subsequent passaging and expansion. These selected clones had an undifferentiated morphology, which was later confirmed by immunofluorescence, and had taken up both the dTOMATO and EGFP constructs; any clone that expressed the tagBFP construct was not expanded as a monoclonal iPS cell line. The selected clones were dissociated using Accutase and were expanded into a well of a 24-well plate before being passaged into two wells of a 12-well plate. At this stage, one of the cell clones was cryopreserved (70% knockout serum replacement (KOSR), Life Technologies, Thermo Fisher Scientific, Waltham, MA, USA; 1867715, 20% E8, 10% DMSO, Sigma-Aldrich, St. Louis, MO, USA; D2438) with 10 μM of the Rho-Kinase inhibitor. The remaining clone was pelleted for PCR-mediated clonal selection using *Mva1* restriction digestion.

### 2.6. PCR Restriction Digest Mediated Clonal Selection

Genomic DNA was extracted from the iPSCs using the Blood and Cell Culture DNA Kit (Qiagen, Hilden, Germany, 13323) according to the manufacturer’s instructions. Amplification of Exon 2 *SNCA* by PCR was performed using the forward primer: CCCCGAAAGTTCTCATTCAA and reverse primer: GCGAATCCGTCGCTGTGCAT giving a 314 bp product. Kit Go Taq^®^ G2 Flexi DNA Polymerase (Promega, Madison, WI, USA; M7805) was used for this reaction with the following program: predenaturation (95 °C; 2 min), 35 cycles of denaturation (95 °C; 30 s), annealing (60 °C; 30 s) and extension (72 °C; 60 s), followed by a final extension (72 °C; 5 min). *Mva1 (BstNI*) (37 °C; 30 min) (ThermoFisher, Waltham, MA, USA; FastDigest #FD0554)) digestion was used to check clonal efficiency. The c.88G>C, p.A30P *SNCA* mutation leads to the creation of a *Mva1* restriction site [4], correction of this mutation results in the undigested product. Following the restriction digest mediated clonal selection, Sanger sequencing was performed on selected cell lines by Eurofins Genomics GmBH (Ebersberg, Germany).

### 2.7. Transposase-Mediated Generation of Footprint-Free Isogenic Cell Lines

Both the dTOMATO-T2A-Puromycin cassette and the EGFP-TA-Puromycin cassette are flanked by PB inverted repeats with a TTAA sequence necessary for their removal [16]. The single cell clones containing the tagBFP-/EGFP+/dTOMATO+ combination were transfected with an mRNA encoding excision-only transposase as previously described [16]. The Stemfect™ RNA Transfection Kit (Stemgent, Cambridge, MA, USA) was used according to the manufacturing instructions; this led to the generation of footprint-free isogenic cell lines. Three sorting steps were undertaken to purify and confirm removal of the biallelic constructs (Supplementary Figure S3).

### 2.8. Trilineage Directed Differentiation

iPSCs were plated onto Geltrex™-coated glass coverslips. Directed in-vitro differentiation to the three germ layers was performed using the Human Pluripotent Stem Cell Functional Identification Kit (R&D Systems, Minneapolis, MN, USA; SC027B) according to the manufacturer’s instructions. Primary antibodies used provided with the kit were: SOX17 (1:1000, R&D Systems, Minneapolis, MN, USA; #963121), OTX2 (1:1000, R&D Systems, Minneapolis, MN, USA; #963273) and BRACHYURY (1:1000, R&D Systems, Minneapolis, MN, USA; #963427). The secondary antibody used was Alexa Fluor 647 Donkey anti-Goat IgG (H+L; 1:1000, Invitrogen, Thermo Fisher Scientific, Waltham, MA, USA; A21447). Cells were fixed, stained and imaged as previously described [27].

### 2.9. Neuronal Differentiation

iPSCs were directly differentiated into a multipotent neuronal progenitor cell (NPC) population using a previously published protocol [29]. The NPCs were used after 10 enzymatic purification passages. Coexpression of the neural stem cell markers: NESTIN and MUSHASHI were used to characterize the cell type [30]. Ventral midbrain dopaminergic neurons were directly differentiated for 30 days from the NPCs stage using the previously published protocol [29]. Coexpression of the ventral midbrain marker FOXA2, the neuronal marker TUJ1 and dopaminergic marker TH was used to characterize the cellular population.

### 2.10. Immunocytochemistry

Cells were fixed, stained and imaged as previously described [27]. Primary antibodies used were: OCT4 (1;200; Santa Cruz Biotechnology, Dallas, TX, USA; sc-5279), NANOG (1:100; Abcam, Cambridge, UK; ab21624), SOX2(Y-17) (1:200; Santa Cruz Biotechnology, Dallas, TX, USA; sc-17320), TRA-1-60 (1:300; Abcam, Cambridge, UK; ab16288), NESTIN (1:500; R&D Systems, MAB1259), MUSHASHI (1:500; Abcam, Cambridge, UK; ab21628), FOXA2 (HNF3β) (RY-7; 1:100; Santa Cruz Biotechnology, Dallas, TX, USA; sc-101060), β-3-Tubulin (TUJ1; 1:200; BioLegend, San Diego, CA, USA; 801201) and TH (1:300; Millipore, Burlington, MA, USA; AB152). Secondary antibodies used were: Alexa Fluor 488 Goat anti-Mouse IgG (H+L; 1:200; Invitrogen, Thermo Fisher Scientific, Waltham, MA, USA; A11029), Alexa Fluor 568 Goat anti-Rabbit IgG (H+L; 1:200; Invitrogen, Thermo Fisher Scientific, Waltham, MA, USA; A11036) and Alexa Fluo 647 Donkey anti-Goat IgG (H+L; 1:200; Invitrogen, Thermo Fisher Scientific, Waltham, MA, USA; A21447). Image analysis was carried out using Zen Blue confocal software (Carl Zeiss AG, Oberkochen, Germany). All images were processed using Adobe Photoshop CS6 (Adobe Inc. San Jose, CA, USA).

### 2.11. Flow Cytometry

Neurons at d30 of differentiation were treated with Accutase and placed in an incubator (37 °C, 5% CO_2_) for 5-10 min. The neurons were dissociated to obtain a single cell suspension before DMEM was added and the cells were centrifuged (300× *g*; 5 min). The neurons were resuspended in 100 μL of PBS. Dropwise, 400 μL of 4% PFA was added to the cell suspension whilst vortexing. The cells were placed in a tube rotator at 4 °C for 15 min. PBS was added and the neurons were spun down (300× *g*; 5 min). The neurons were resuspended in PBS and centrifuged a further two times to ensure all PFA had been removed from the cellular suspension. Cells were then resuspended in 300 μL of Saponin buffer (0.05% Saponin/1% BSA/PBS), and placed in a tube rotator at 4 °C for 30 min. Cells were then resuspended (800 g; 5 min) with primary antibodies and placed in a tube rotator at 4 °C for 30 min. The cells were spun down, resuspended three times in 5% BSA/PBS and then resuspended with secondary antibodies (1:100) PBS/5% BSA. The cells were then placed in a tube rotator at 4 °C for 30 min, centrifuged and resuspended three times in PBS before analyzed on the BD LSRFortessa™ cell analyzer (BD Biosciences, Franklin Lakes, NY, USA) in 300 μL of PBS. Primary antibodies used were: NESTIN (1:100), MUSHASHI (1:100), TH (1:100) and TUJ1 (1:200). Primary antibodies used as negative controls were: FLAG (1:100; Sigma-Aldrich, St. Louis, MO, USA; F1804) and GFP (1:100; Santa Cruz Biotechnology, Dallas, TX, USA; sc-8334). For NESTIN/MUSHASHI, TUJ1 and TH/TUJ1 cell counts, FlowJo™ software (FlowJo for Windows, Version V10, 2019, Becton, Dickinson and Company, Franklin Lakes, NY, USA) was used for gating and processing of the samples.

### 2.12. Protein Immunoblotting

The protein immunoblotting cells pellets were extracted using RIPA buffer (Tris HCl pH 7.4, (50 mM); NaCl (150 mM); Triton-X-100 (1%); sodium deoxylcholate (0.5%); SDS (0.1%); EDTA (1 mM); Tris-HCl (50 mM) and NaCl (150 mM)) plus 1 tablet of cOmplete™ proteinase inhibitor cocktail (Roche, Basel, Switzerland) per 20 mL of RIPA buffer. Polyacrylamide gels (10%) were blotted by dry transfer on 0.2 μM nitrocellulose membranes using the iBlot™ 2 Gel Transfer Device (ThermoFisher, Waltham, MA, USA). Primary anti-ALPHA-SYNUCLEIN (1:1000, BD Transduction Laboratories, Franklin Lakes, NY, USA; 610787), TH (1:1000) and -β-ACTIN (1:20,000; Cell Signaling Technology, Danvers, MA, USA; 3700S) antibodies were probed overnight followed by incubation for 1 h with secondary antibodies conjugated to HRP (Invitrogen, ThermoFisher Scientific, Waltham, MA, USA). Densitometry was performed using ImageJ [31] and protein normalized to β-actin.

### 2.13. RT-qPCR

RT-qPCR was used as previously outlined [27]. Briefly, total RNA was extracted from d30 neurons using the RNEasy mini kit (Qiagen, Hilden, Germany) according to the manufacturer’s instructions. Transcriptor High Fidelity cDNA Synthesis Kit (Roche, Basel, Switzerland) was used to synthesize cDNA. *SNCA* gene expression was quantified by Multiplex qPCR using the LightCycler^®^ 480 Probes Master kit (Roche, Basel, Switzerland) run on the LightCycler^®^ 480 (Roche, Basel, Switzerland). Hydrolysis probes against *SNCA* (Hs01103383_m1), *ACTB* (Hs03023880_g1) and *GAPDH* (Hs02758991_g1) were used with gene expression normalized to *ACTB* or *GAPDH*. Total RNA from patient-derived fibroblasts were used as a negative control [27]. Each sample and primer set was run in triplicates and relative expression levels were calculated using the ΔΔCt method.

### 2.14. Chromosomal Analysis

Molecular karyotyping and identity analysis was performed on the iPS clones at Life&Brain GmBH (Bonn, Germany) using the Illumina BeadArray HumanOmni2.5Exome-8 BeadChip v1.3 on the Illumina iScan (Serial Number: N263) scanner (Illumina Inc. San Diego, CA, USA). A genotype analysis was performed using GenomeStudio V2.0.2 with a copy number analysis undertaken using the CNV-Partition V3.2 (Illumina Inc. San Diego, CA, USA). Copy number events were reported if larger than 350,000 base pairs. The method overviewing the high-resolution whole sequence genomic profiling technology that was used in this study has been previously described [32]. The molecular karyotyping report for each iPS clone analyzed are available with the authors and can be made available upon reasonable request.

### 2.15. Computer Code, Software and Licensing

Fluorescence microscopy images were acquired on an Opera QEHS spinning disc microscope (PerkinElmer Inc., Waltham, MA, USA) using a 10× air objective with numerical aperture 0.4. Blue, green and red fluorescent channels were acquired simultaneously. The sample was excited with 405 nm, 488 nm and 561 nm lasers. Blue emission was detected behind a 450/50 bandpass filter, green emission behind a 520/35 filter and red emission behind a 600/40 filter. The camera binning for all channels was 2.

Image analysis was performed in Matlab 2017b (MathWorks, Natick, MA, USA). For the classification of clones, whole well mosaic images were segmented and classified with a custom algorithm: Briefly, the red channel was low pass filtered with a Gaussian kernel of size 21 and standard deviation 7, and thresholded (RedPositiveMask = RedLP > 125). The green and red channels were low pass filtered with Gaussian kernels of size 60 and standard deviation 20 and thresholded (GreenPositiveMask = GreenLP > 200, BluePositiveMask = BlueLP > 200). After segmentation, wells containing a clone with an area bigger than 20,000 pixels were classified as Blue, Red, RedGreen or Negative according to the area proportion (AP) per channel. Area proportion is defined as the count of pixels in a channel-specific mask divided by the count of pixels in the clonal region of interest, which is defined by the Boolean OR operation between GreenPositiveMask and RedPositiveMask. Briefly, the class Red is defined as APred > 0.9 and APgreen < 0.1. The class Green is defined as APred < 0.1 and APgreen > 0.9, the class RedGreen is defined as APred > 0.1 and APgreen > 0.1 and all remaining clones are classified as Negative.

The figure created for the graphical abstract was generated using images from Servier Medical Art Commons Attribution 3.0 Unported License. (http://smart.servier.com). Servier Medical Art by Servier is licensed under a Creative Commons Attribution 3.0 Unported License.

## 3. Results

### 3.1. Single-Cell Sorting of Gene-Edited iPSC Clones

**Figure 3:**
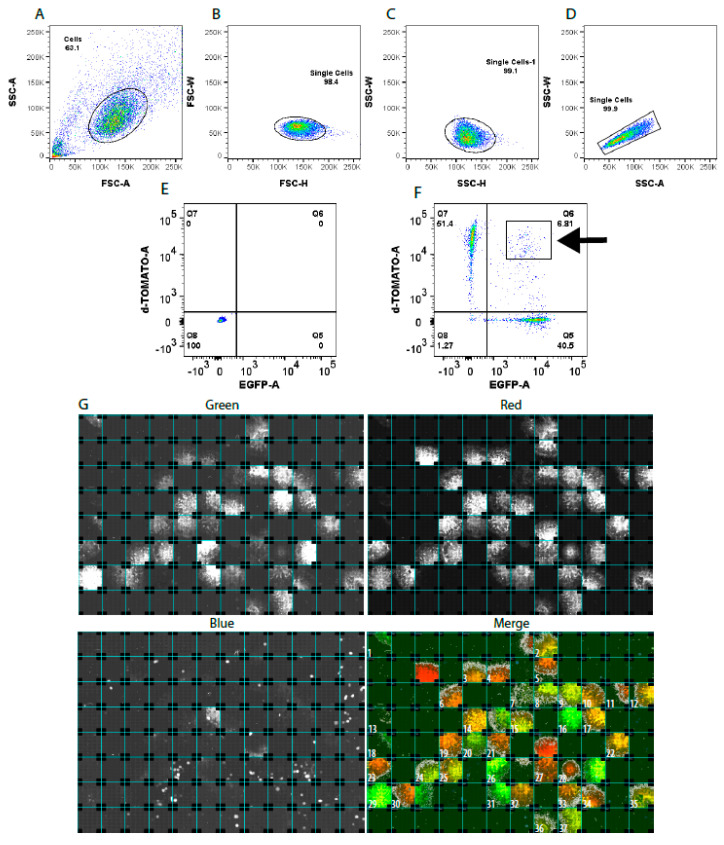
Sorting of single-cell isogenic induced pluripotent stem cells (iPSCs). (**A**) Selection of a live cell population and **(B**–**D**) doublet-discrimination. (**E**) Generation of negative sorting gates using untransfected iPSCs. (**F**) Single-cell fluorescence activated cell sorting (FACS) sorting of dTOMATO^+^/EGFP^+^ cells with restrictive gating (black arrow) into a 96 well plate. (**G**) High content screening of single-cell sorted plate showing the green, red, blue and merged channels.

The pluripotent iPSC line A30P-4 (unique identifier HIHDNDi001-B) [27], derived from the p.A30P *SNCA* patient underwent electroporation with biallelic targeting plasmids containing wild-type homology sequence attached with two different fluorescent reporters, a red fluorescent protein (dTOMATO) and a green fluorescent protein (EGFP). Biallelic editing using two fluorescent constructs provides the certainty of generating the homozygous wild-type isogenic cell line. Using FACS, double-discrimination for dead cells, cell clumps and doublets were used with strict gating (Figure 3A–D). The negative control used was the iPSC line without transfection (Figure 3E), a representative plot of the sorted dTOMATO^+^/EGFP^+^ cells is shown in Figure 3F. Outside of the homology arm there is a blue fluorescent protein (tagBFP) that allows for the identification of random integration events, which was removed by prior cell sorting (Supplementary Figure S1). The box and arrow in Figure 3F represents the gating used to sort the single-cells into the 96-well plate. Once fluorescence was evident in cells, they were screened using the OPERA HCS with Matlab (version 2017b, MathWorks, Natick, MA, USA). The in-house developed image analysis algorithm (https://doi.org/10.17881/lcsb.kcqg-tr55) automates the segmentation of the cellular structure across three fluorescent channels and shows a merged image, with the clones numbered (Figure 3G). The use of HCS technology allows the operator to discriminate based on the presence of a single fluorescent construct only, a BFP^+^ cell, iPS morphology and cell doublets from improper FACS gating.

### 3.2. Restriction-Digest Mediated Selection of Single-Cell Lines

**Figure 4:**
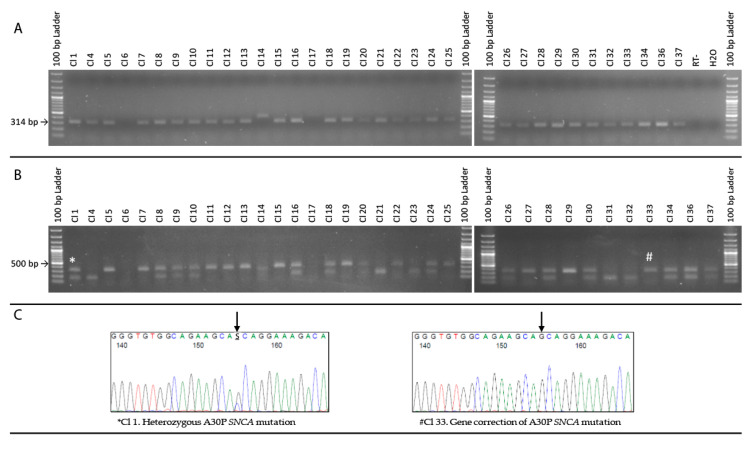
Restriction digest mediated clonal selection. (**A**) PCR amplification of single-cell clones. (**B**) *Mva1* restriction digest post PCR amplification. *Double-band and #Unedited restriction digestion. (**C**) Sanger sequencing of the amplified sequence. Black arrow signifies the location of the c.88G>C *SNCA* mutation.

Following the positive selection and expansion of the monoclonal iPS cell lines using FACS and HCS, PCR amplification and restriction digestion was the next set of selection criteria used for quality control of the single-cell iPS cell lines. Of the 37 different monoclonal iPSC cell lines amplified (Figure 3G), 34 of those cell lines were successfully passaged and cryopreserved. Specific primers were used to amplify Exon 2 of the *SNCA* gene leading to a 314 bp product, a further three clones (Cl. 6, Cl. 14 and Cl.17) failed in the initial PCR amplification (Figure 4A), with Cl. 14 having introduced an insertion of the sequence (Supplementary Figure S2). The c.88G>C p.A30P *SNCA* heterozygous mutation leads to the generation of an *Mva1* restriction site. Conversely, if the heterozygous mutation has been repaired by HDR and gene-corrected, there should be no *Mva1* restriction site and therefore no digested product. Of the clones that are putatively isogenic, there are three distinct PCR products (Figure 4B). These are shown in Figure 3B as a double-band (*), a lower band of approximately 100 bp or an undigested PCR product (#). Using Sanger sequencing (Figure 3C) we confirmed that the clones, which had the double-band retained the heterozygous c.88G>C mutation and were not correctly edited. The iPS single-cell clones with the undigested PCR product at 314 bp showed the successful generation of gene-corrected *SNCA* cell lines.

A list detailing the CRISPR-Cas9 mediated monoclonal isogenic patient-derived iPSC lines generated in this study, including cell-line validation criteria is shown in Table 1. 

### 3.3. Characterization of Single Cell Clones

Three cell lines of the gene-corrected isogenic cell lines (Cl. 5, Cl. 13 and Cl. 33) were selected at random and transfected with an mRNA encoding the excision-only variant of the piggyBac transposase to remove the fluorescent constructs [20,33]. The subsequent dTOMATO^-^/EGFP^-^ cell population was purified by three cell sorting steps (Supplementary Figure S3). The transposon-mediated excision efficiency is shown in Supplementary Table S1. The gene-corrected footprint-free isogenic cell lines were then karyotyped as part of the validation procedure. The gene-corrected cell lines Cl. 13 and Cl. 33 were karyotypically normal, had a stable genotype and passed this validation step, Cl. 5 had a deletion in Chromosome 4 and did not pass the validation step. All molecular karyotypes using a single nucleotide polymorphism (SNP) analysis are shown in Supplementary Figures S4–S6.

**Figure 5:**
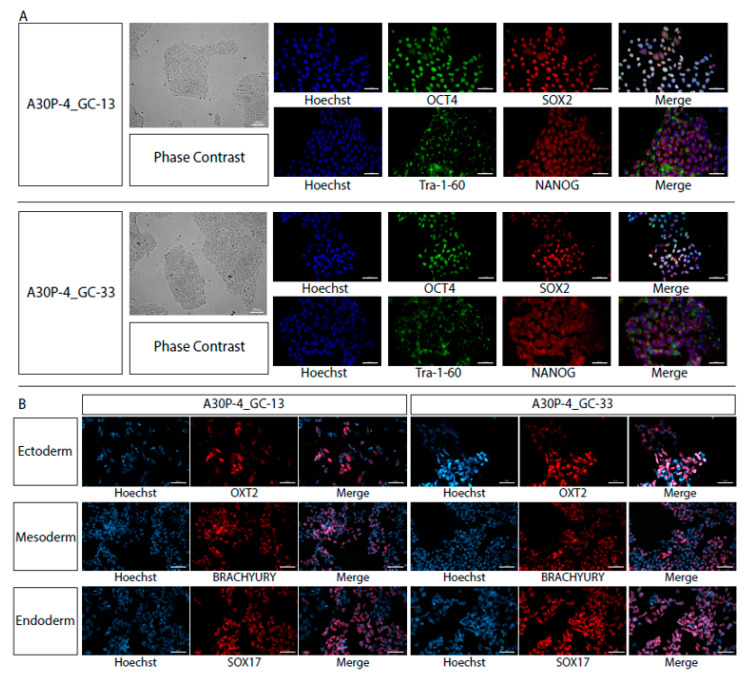
iPS cell line characterization. (**A**) Pluripotency characterization of the three single-cell isogenic lines generated in this project. Phase contrast images of pluripotent colonies, image taken using a 5× objective. Scale bar represents 100 μM. Antibody specific pluripotency marker expression of OCT4/SOX2 and NANOG/TRA-1-60. Images taken using a 25× objective, scale bar is 50 μM. (**B**) Directed differentiation to the three embryonic germ layers using antibodies specific to the ectoderm (OTX2), endoderm (SOX17) and mesoderm (BRACHYURY). Images taken using a 25× objective, scale bar is 50 μM.

The de-novo monoclonal gene-edited iPS cell lines were then characterized and validated by their pluripotency ability. The iPS colonies displayed stem cell morphology and had positive marker staining for: OCT4, SOX2, NANOG and TRA-1-60 at the protein level (Figure 5A). The iPS cell lines also retained the in-vitro ability to directly differentiate into the three embryonic germ layers defined by specific marker expression specific to that lineage: ectoderm (OTX2), endoderm (SOX17) and mesoderm (BRACHYURY; Figure 5B).

### 3.4. Generation and Characterization of Neural Precursor Cells Reveals Reduced SNCA Expression in the Gene-Corrected Control NPCs

**Figure 6:**
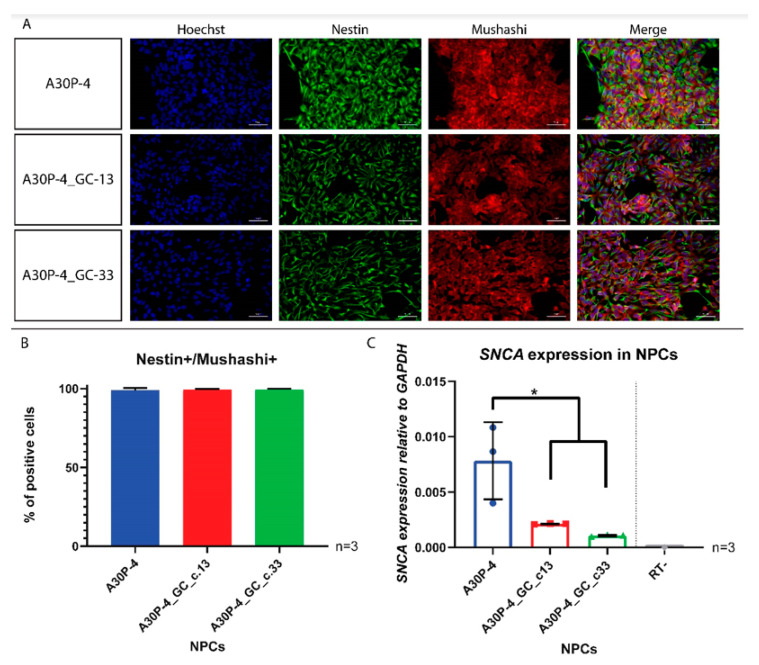
Characterization of neural precursor cells (NPCs) and quantification of SNCA expression. (**A**) Immunofluorescence of NESTIN and MUSHASHI antibody staining. Scale bar is 50 μM. (**B**) Quantification of NESTIN+/MUSHASHI+ NPCs by flow cytometry (*n* = 3). (**C**) Quantitative gene expression of SNCA in NPCs (*n* = 3). An ordinary one-way ANOVA was performed using the Tukey post hoc multiple comparison test. *p < 0.05.

In order to validate the de-novo gene-corrected monoclonal cell lines we conducted a direct head-to-head comparison with that of the founder patient line: A30P-4, which harbors the heterozygous c.88G>C mutation in the *SNCA* gene leading to the pathogenic p.A30P form of the alpha-synuclein protein. We generated neural precursor cells (NPCs) using the protocol elsewhere described [29], these multipotent neural stem cells exhibit immortal expansion and have been extensively used as a model of neurodegenerative disease and in drug discovery [34,35]. Coexpression of the neural stem cell markers NESTIN and MUSHASHI was used to validate and compare the multipotent cellular fate. All cell lines show positive expression of both NESTIN and MUSHASHI by immunocytochemistry (Figure 6A), quantification performed using flow cytometry shows that approximately all of the cells per derived cell line are NESTIN+/MUSHASHI+ (Figure 6B), gating and negative control shown in Supplementary Figure S7. Direct comparison of the multipotent NPCs revealed that both of the gene corrected clones had reduced *SNCA* expression compared to the NPCs carrying the p.A30P alpha-synuclein mutation (Figure 6C).

### 3.5. Directed Differentiation and Characterization of Midbrain Dopaminergic Neurons Reveals Reduced SNCA Expression and Alpha-Synuclein Level in the Gene-Corrected Control Neurons

**Figure 7:**
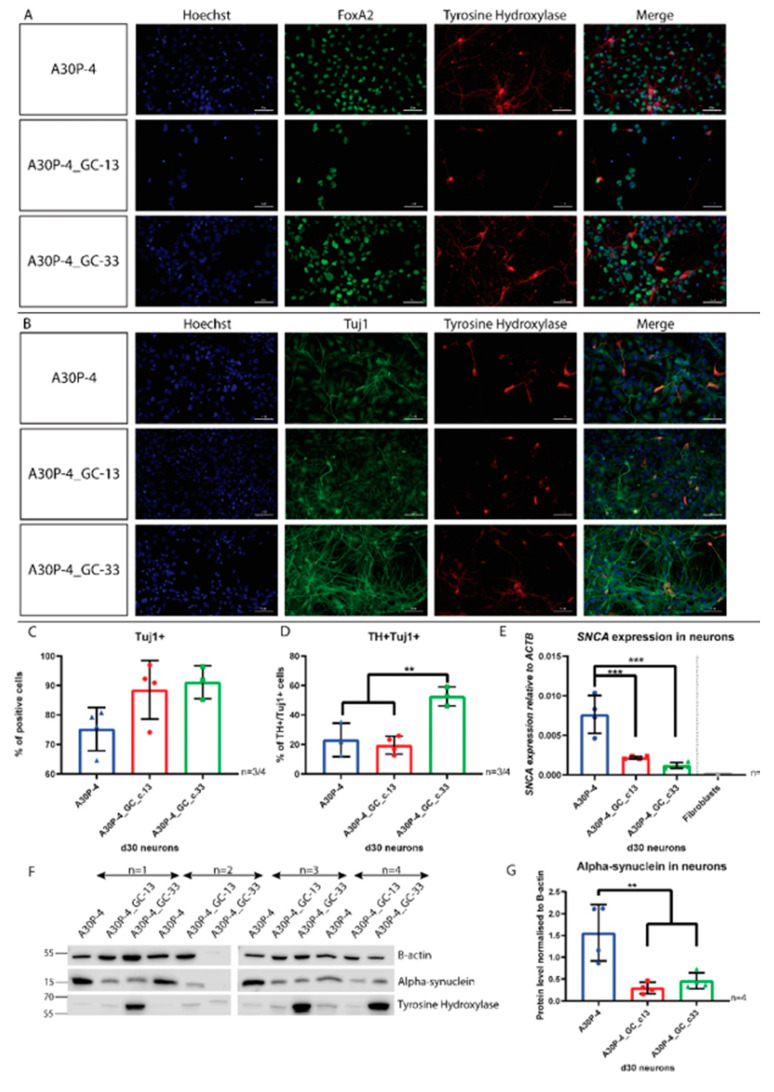
Characterization of vmDANs and quantification of SNCA expression and alpha-synuclein level. (**A**,**B**) Immunofluorescence of FOXA2, TH and TUJ1. Scale bar is 50 μM. (**C**) Quantification by flow cytometry of TUJ1 neurons (*n* = 3/4), and (**D**) TH+/TUJ1+ neurons (*n* = 3/4; ANOVA, **p < 0.01). (**E**) Relative quantification of SNCA expression in vmDANs (*n* = 4), (ANOVA, ***p < 0.001). (**F**) Protein immunoblot showing alpha-synuclein and TH protein level in vmDANs. (**G**) Quantification of alpha-synuclein protein (*n* = 4; ANOVA, **p < 0.01). For all statistical analyses, ordinary one-way ANOVA’s were performed using the Tukey post hoc multiple comparison test.

To assess the p.A30P mutation in a disease relevant cellular model, we directly differentiated the NPCs into vmDANs in order to recapitulate the neurons degenerated in PD. Characterization of these neurons in each cell line after 30 days of differentiation shows immunopositive staining for the ventral midbrain marker FOXA2, the neuronal marker TUJ1 and the rate-limiting enzyme used to identify dopaminergic neurons TYROSINE HYDROXYLASE (TH; Figure 7A,B).

Quantification of the neurons using flow cytometry revealed no significant differences between the cell lines in their capacity for neuronal differentiation (Figure 7C). We then quantified the amount of dopaminergic neurons within our neuronal population. The negative controls and gating used are shown in Supplementary Figure S8. Here we found differences between the gene-corrected clones, where the gene-corrected clone 33 generated a significantly higher number of TH+ neurons and an increased amount of TH protein than the other cell lines (Figure 7D,G). As this difference was not found in the other gene-corrected clone, it can be inferred that it is the variability between cell lines that is responsible for this difference. This example of intraclonal variation is a limitation within the field of iPSC-based disease modeling that has been reported elsewhere [36,37,38]. Relative quantification of gene expression using RT-qPCR confirmed our finding of reduced *SNCA* expression in the NPCs with approximately a 3-fold reduction found in the neurons (Figure 7E). To validate the difference in gene expression against the translated protein we performed a Western blot and also found an approximate 3-fold reduction in the alpha-synuclein protein in the two gene-corrected controls compared to the p.A30P patient neurons (Figure 7F,G).

### 3.6. Relative Quantification across Cell Types Reveal Reduced SNCA Gene Expression in the Gene-Corrected Controls

**Figure 8:**
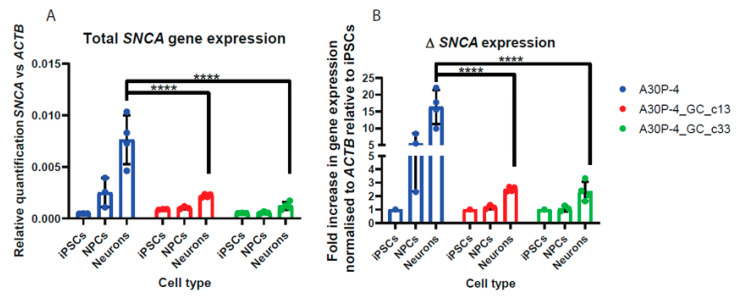
Change in SNCA expression in iPSCs, NPCs and neurons. (**A**) Relative quantification of SNCA mRNA expression across multiple cell lines. (**B**) Fold change in gene expression relative to iPSCs. An ordinary 2-way ANOVA statistical test was performed with Tukey’s multiple comparison post-hoc test. ****p < 0.0001.

To confirm that the gene-corrected patient-derived cell lines have reduced *SNCA* expression than the A30P patient, we compared the *SNCA* expression in iPSCs, NPCs and neurons. We found that specifically neurons carrying the p.A30P mutation had excess levels of *SNCA* expression (Figure 8A). Furthermore, there was approximately a 15-fold increase in SNCA expression from A30P-4 neurons compared to the iPSCs, this compares against a 3-fold increase in SNCA expression in both gene-corrected controls (Figure 8B). Thereby, there was a 5-fold increase in neuronal SNCA expression in the A30P patient compared to the gene-corrected controls (Figure 8B).

## 4. Discussion

This research article is the first to show gene-corrected isogenic cell lines from a PD patient carrying the mutation encoding p.A30P in the *SNCA* gene. Gene-correction of the patient iPSCs carrying the pathogenic p.A53T *SNCA* mutation and triplication of the *SNCA* gene locus have been previously published [24,39,40].

This study is the first to generate patient-derived gene-corrected single-cell isogenic iPS cell lines and is a novel approach within the literature using a quadruple selection strategy (QSS), to select, screen and validate these single-cell clones using genotyping and pluripotency characterization. The use of the QSS: antibiotic resistance; single-cell sorting and HCS; PCR amplification and restriction digest-mediated selection and Sanger sequencing, to select clones is additionally an approach that has the scope to be scaled up for high-throughput mediated generation of isogenic iPS cell lines. The use of the QSS will substantially reduce the time required to generate an isogenic cell line, in part by eliminating every other non-edited or incorrectly edited cell from the edited population. Sorting and screening multiple 96-well plates using HCS further reduces the time that the operator needs to spend manually checking and validating each colony/well through the microscope. PCR-mediated restriction digestion has long been used successfully in gene editing to validate generated patient-derived isogenic iPS clones [24] and introducing silent mutations or utilizing the existing mutation within the sequence of the pre-edited cell line is one of the most effective strategies in validating the generated patient-derived isogenic iPS clone.

Using the QSS strategy to generate the single-cell isogenic clones gives an efficiency of 35.30%—12 out of the 34 *Mva1* undigested clones were correctly edited (Table 2), which compares favorably to the standard isogenic generation rate of 1–5% [18,25]. However, it must be noted that without the sorting of the edited cells into single-cell clones, this efficiency would be markedly lower. The remaining 64.70% of sorted cells that had expressed both wild-type sequences shown by the presence of the respective dTOMATO and EGFP constructs were either incorrectly edited or unedited in the sequenced region. Without the single-cell isogenic method of selection, a heterozygous polyclonal mix of correctly and differentially edited cells would have been generated and not a bona-fide gene-corrected patient-derived isogenic iPS cell line. Moreover, the polyclonal cell line would not have passed the *Mva1* undigested restriction-digest validation step and the line would have not been generated, underlying the stringency of the validation.

The plated recovery rate of the single-cell iPS clones was 19.27%, and this already contained the correct fluorescent combination of dTOMATO^+^/EGFP^+^/tagBFP^-^ (Table 2). A concern in the literature regarding the dissociation to single-cells and single-cell iPS culture is that there is a strong selective pressure for iPSCs to adapt that could lead to potential genomic abnormalities [41,42]. Changes in copy number variation (CNV) were used to sequence the genome and validate the isogenic cell lines after transposase-mediated excision of the construct. Two of the three single-cell isogenic cell lines selected at random had no genotypic abnormalities although the third did. However as this was due to a deletion on chromosome 4 (Table 1) it is likely this was mediated by gene editing as opposed to a cell culture acquired abnormality [43].

The generation of the two characterized gene-corrected monoclonal iPS cell lines into NPCs and the subsequent characterization of the multipotent neural progenitor cells revealed no difference in the cellular composition of the precursor population. However, even at the neural progenitor stage there is an increase in *SNCA* expression in the patient line containing the p.A30P alpha-synuclein mutation, implicating a role for alpha-synuclein in neurodevelopment, a link that has been already made indirectly [44], and directly with neuronal differentiation and maturity impaired in patient-derived neurons carrying a triplication of the *SNCA* locus [45].

The subsequent differentiation of the NPCs into post-mitotic ventral midbrain neurons show that *SNCA* expression and endogenous alpha-synuclein protein level in the A30P mutant is also higher compared to the gene-corrected controls. This increased alpha-synuclein levels is independent to the amount of TH positive neurons obtained in the cellular differentiations derived from each cell line, reflecting of an increase in alpha-synuclein in the A30P patient across differentiated neuronal subtypes.

Multiple studies have shown that excess alpha-synuclein is pathogenic, the increased *SNCA* gene dosage is causative for PD and the triplication is clinically more severe than the duplication [8,9,10,11,39,40,46]. Furthermore, it is well established that the heterozygous point mutations in *SNCA*: p.A30P, p.A53T, p.A53E, p.E46K and p.G51D, lead to misfolding and aggregation of the monomeric alpha-synuclein protein [47,48]. It was however unclear, which impact the point mutations had on the endogenous level of *SNCA* expression and alpha-synuclein protein. This study shows in a patient-derived model containing an autosomal dominant mutation in *SNCA* that the p.A30P alpha-synuclein mutation has significantly increased levels of *SNCA* expression and endogenous monomeric protein as compared to its isogenic controls. Genetic correction of the patient-derived p.A53T *SNCA* mutation by Ryan and colleagues also found increased levels of alpha-synuclein protein in the patient neurons in comparison to the gene-corrected isogenic control, although no quantifiable differences in RNA levels were identified [39], however, this could also point to the increased stability of the p.A53T form of the alpha-synuclein protein [49].

In contrast, a reduced level of *SNCA* mRNA has been reported in individuals harboring either p.A30P or p.A53T *SNCA* point mutations using lymphoblastoid cell lines [50,51]. The individuals with reduced mRNA expression had a severe clinical phenotype, yet the mildly affected and asymptomatic individuals had no difference in *SNCA* mRNA expression, associating increased clinical PD severity with reduced *SNCA* expression [50]. However, there are significant differences between these studies and ours that require consideration. Principally, Epstein–Barr virus- (EBV-) immortalized lymphoblastoid cell lines are not a disease-specific cell type when we consider that PD is characterized by the loss of vmDANs of the SNc, it is also necessary to consider that the functional role of the alpha-synuclein protein may diverge between those cell types. Additionally, there is individual variability in alpha-synuclein levels, which is influenced by genetic variability in the promoter and 3′ region of the *SNCA* gene [52], here the availability of isogenic cell lines allow the assessment of alpha-synuclein specific to that individual.

The relative maturity of the patient-derived ventral midbrain neurons is also of interest. *SNCA* expression increases during neuronal differentiation [45], and in our model we detected the functional protein 30 days after directed neuronal differentiation using Western blot. However, it is unclear if the alpha-synuclein level will continue to rise with increased neuronal maturity. The neurons carrying the A30P mutation could either have increased levels of alpha-synuclein, or just increased levels of alpha-synuclein at the time-point 30 days after directed neuronal differentiation, a longer time-course experiment would provide a deeper understanding of SNCA expression and neuronal maturity. Furthermore, a single-cell analysis of TH-only neurons or sorting the dopaminergic neurons from a mixed neuronal culture to assess SNCA expression will provide further information on the specifically vulnerable vmDANs carrying the pathogenic p.A30P mutation that degenerate in PD [53].

One of the benefits of the monoclonal gene-corrected cell lines is that it has allowed for the distinction between cell lines that have a high differentiation capacity to dopaminergic neurons and those that do not without seeking to mislabel this as a phenotype that can be misleading for the field. Consequently, differentiation of multiple clones against their founder cell line and significant differences from across all of those of clones’ vs. that founder line will lead to a more thorough indication of true phenotypic difference thus benefiting disease modeling with future implications for drug discovery.

## 5. Conclusions

The generation of gene corrected isogenic cell lines is critically important in understanding the mechanism of how a pathogenic disease variant leads to the disease. Establishing a single-cell gene corrected isogenic as opposed to a heterozygous polyclonal isogenic is the next step in this research landscape. What we have shown is that the use of the QSS that includes the single-cell sorting and HCS easily allows the researcher to generate, validate and isolate the gene-corrected single-cell isogenic clones from the CRISPR-Cas9 correctly edited, incorrectly edited and non-edited heterozygous cellular population, improving on a technically challenging, variably efficient and time-consuming process. As proof of concept we have shown that the c.88G>C heterozygous mutation in *SNCA* when gene-corrected leads to a reduction in the *SNCA* expression and relative alpha-synuclein protein level by approximately a third. Implications for PD research would be to investigate strategies to reduce alpha-synuclein level along those parameters.

## Figures and Tables

**Table 1:** List of CRISPR-Cas9 mediated single-cell isogenic patient-derived iPSC clones generated in this study with cell line validation criteria.

Expanded Single Cell Clones ^1^	PCR Test	*Mva1* Test Digest	Sanger Sequencing	Construct Excision and Karyotyping
A30P-4 edited clone 1	314 bp	Digested: Double-band	Not Performed	
A30P-4 edited clone 4	314 bp	Digested: Lower bp product	Not Performed	
A30P-4 edited clone 5	314 bp	Undigested: 314 bp product	Isogenic: Gene-corrected p.A30P mutation	Chr 4: Deletion
A30P-4 edited clone 6	No product	No product	Not Performed	
A30P-4 edited clone 7	314 bp	Undigested: 314 bp product	Not Performed	
A30P-4 edited clone 8	314 bp	Digested: Double-band	Not Performed	
A30P-4 edited clone 9	314 bp	Digested: Double-band	Not Performed	
A30P-4 edited clone 10	314 bp	Digested: Double-band	Not Performed	
A30P-4 edited clone 11	314 bp	Undigested: 314 bp product	Not Performed	
A30P-4 edited clone 12	314 bp	Undigested: 314 bp product	Not Performed	
A30P-4 edited clone 13	314 bp	Undigested: 314 bp product	Isogenic: Gene-corrected p.A30P mutation	Normal karyotype
A30P-4 edited clone 14	416 bp	Digested	102 bp insertion error	
A30P-4 edited clone 15	314 bp	Undigested: 314 bp product	Not Performed	
A30P-4 edited clone 16	314 bp	Digested: Double-band	Not Performed	
A30P-4 edited clone 17	No product	No product	102 bp insertion error	
A30P-4 edited clone 18	314 bp	Digested: Double-band	Not Performed	
A30P-4 edited clone 19	314 bp	Undigested: 314 bp product	Isogenic: Gene-corrected p.A30P mutation	
A30P-4 edited clone 20	314 bp	Digested: Double-band	Not edited: Heterozygous p.A30P *SNCA* mutation	
A30P-4 edited clone 21	314 bp	Digested: Lower bp product	Not Performed	
A30P-4 edited clone 22	314 bp	Undigested: 314 bp product	Not Performed	
A30P-4 edited clone 23	314 bp	Digested: Lower bp product	Not Performed	
A30P-4 edited clone 24	314 bp	Digested: Double-band	Not Performed	
A30P-4 edited clone 25	314 bp	Undigested: 314 bp product	Not Performed	
A30P-4 edited clone 26	314 bp	Digested: Double-band	Not Performed	
A30P-4 edited clone 27	314 bp	Undigested: 314 bp product	Not Performed	
A30P-4 edited clone 28	314 bp	Digested: Double-band	Not Performed	
A30P-4 edited clone 29	314 bp	Undigested: 314 bp product	Isogenic: Gene-corrected p.A30P mutation	
A30P-4 edited clone 30	314 bp	Digested: Double-band	Not Performed	
A30P-4 edited clone 31	314 bp	Digested: Lower bp product	Not Performed	
A30P-4 edited clone 32	314 bp	Digested: Lower bp product	Not Performed	
A30P-4 edited clone 33	314 bp	Undigested: 314 bp product	Isogenic: Gene-corrected p.A30P mutation	Normal karyotype
A30P-4 edited clone 34	314 bp	Digested: Double-band	Not Performed	
A30P-4 edited clone 36	314 bp	Digested: Double-band	Not Performed	
A30P-4 edited clone 37	314 bp	Digested: Double-band	Not Performed	

^1^ A30P-4 edited clones 2, 3 and 35 did not survive iPS passaging.

**Table 2:** The summary of each stage of the gene-corrected p.A30P *SNCA* single-cell isogenic cell lines generated.

	Sorted Single-Cell Clones ^1^	Expanded Single-Cell Clones	PCR Amplification	*Mva1* Undigested	Sequenced ^2^	Normal Genotype ^3^
Absolute numbers	37/192	34/37	31/34	12/34	5/5	2/3
Efficiency	19.27%	91.89%	91.18%	35.30%	100%	66.67%

^1^ Only the yellow clones expressing both the dTOMATO^+^/EGFP^+^ constructs were amplified. ^2^ Of the cell lines that passed the *Mva1* restriction digest criteria, five were chosen at random for Sanger sequencing.^3^ Of the five cell lines that were successfully sequenced, three were chosen at random for genotyping.

## Supplementary Materials

The following are available online at https://www.mdpi.com/2073-4409/9/9/2065/s1. Figure S1: Removal of tagBFP^+^ cells using FACS prior to sorting the dTOMATO^+^/EGFP^+^ transfected cells. Figure S2: 102 bp sequence insertion in Cl. 14. Figure S3: Transposase removal of biallelic constructs using FACS and validation of footprint-free isogenic cell lines. Figure S4: Molecular karyotype of the single-cell gene-corrected cell line clone 5. Figure S5: Molecular karyotype of the single-cell gene-corrected cell line clone 13. Figure S6: Molecular karyotype of the single-cell gene-corrected cell line clone 33. Figure S7: Gating used to assess Nestin/Mushashi coexpression by a flow cytometry analysis. Figure S8: Gating used to analyze neuronal expression by a flow cytometry analysis. Table S1: Transposons-mediated excision efficiency.
