# Supplementary material for: Using High-Content Screening to Generate Single-Cell Gene-Corrected Patient-Derived iPS Clones Reveals Excess Alpha-Synuclein with Familial Parkinson’s Disease Point Mutation A30P
Source: Cells. 2020 Sep 10;9(9):2065. doi: 10.3390/cells9092065 (PMC7564375; doi:10.3390/cells9092065)
Supplement: Supplementary file 1 [file cells-09-02065-s001.zip › Supp Figures/Supp. Figure 1.pdf]

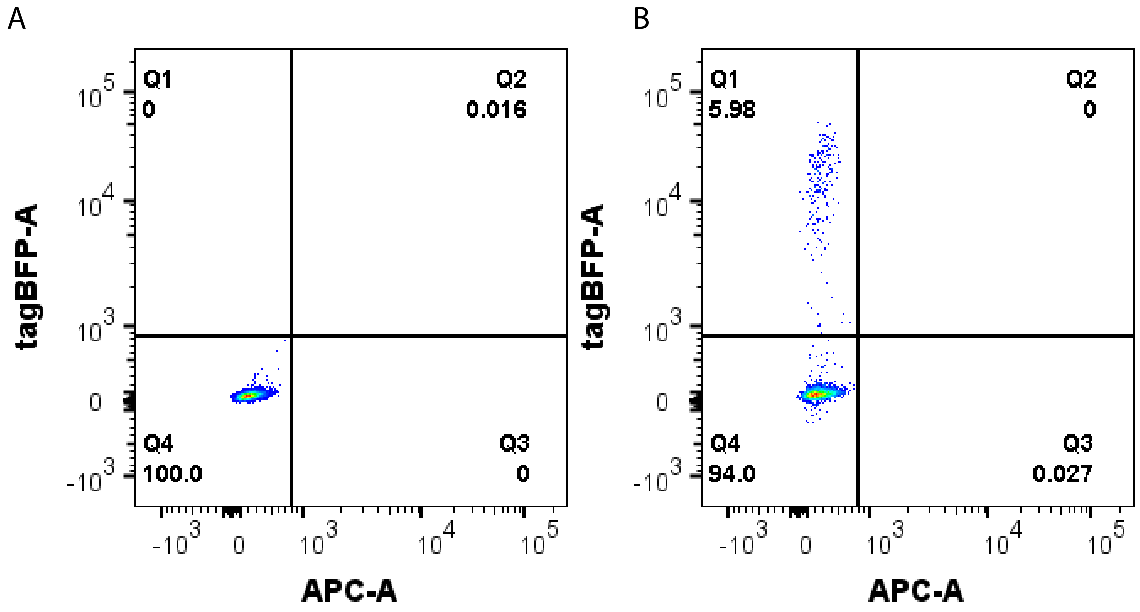

Supplementary Figure 1: Removal of tagBFP+ cells using FACS prior to sorting the dTOMATO+/EGFP+ transfected cells. (A) Untransfected control used to set the gates. (B) tagBFP+ and tagBFP- cells, black arrow and gate indicates the tagBFP negative fraction.
