# Supplementary material for: Using High-Content Screening to Generate Single-Cell Gene-Corrected Patient-Derived iPS Clones Reveals Excess Alpha-Synuclein with Familial Parkinson’s Disease Point Mutation A30P
Source: Cells. 2020 Sep 10;9(9):2065. doi: 10.3390/cells9092065 (PMC7564375; doi:10.3390/cells9092065)
Supplement: Supplementary file 1 [file cells-09-02065-s001.zip › Supp Figures/Supp. Figure 2.pdf]

```
Seq_1  1     -CACCACGTGTGCTAGTGTGGTGTAAGGATTCATTAGCCATGGATGTATTCATGAAAGGA  59
             ###||##|#|||##||||||||||||||||||||||||||||||||||||||||||||||
Seq_2  1     CACCCCAGGGTGG-AGTGTGGTGTAAGGATTCATTAGCCATGGATGTATTCATGAAAGGA  59

Seq_1  60    CTTTCAAAGGCCAAGGAGGGAGTTGTGGCTGCTGCTGAGAAAACCAAACAGGGTGTGGCA  119
             ||||||||||||||||||||||||||||||||||||||||||||||||||||||||||||
Seq_2  60    CTTTCAAAGGCCAAGGAGGGAGTTGTGGCTGCTGCTGAGAAAACCAAACAGGGTGTGGCA  119

Seq_1  120   GAAGCAGCAGGAAAGACAAAAGAGGGTGTTCTCTATGTAGGTAGGTAAACCCCAAATGTC  179
             ||||||#|||||||||||||||||||||||||||||||||||||||||||||||||||||
Seq_2  120   GAAGCACCAGGAAAGACAAAAGAGGGTGTTCTCTATGTAGGTAGGTAAACCCCAAATGTC  179

Seq_1  180   AGTTTGGTGCTTGTTCATGA----------------------------------------  199
             ||||||||||||||||||||########################################
Seq_2  180   AGTTTGGTGCTTGTTCATGATAAATAAACCTCGATATACAGACCGATAAAACACATGCGT  239

Seq_1  200   ------------------------------------------------------------  199
             ############################################################
Seq_2  240   CAATTTTACGCATGATTATCTTTAACGTACGTCACAATATGATTATCTTTCTAGGGTTAA  299

Seq_1  200   ---GTGATGGGTTAGGATAATCAATACTCTAAATGCTGGTAGTTCTCTCTCTTGATTCAT  256
             ###|||||#|||||||||||||||||||||||||||||||||||||||||||||||||||
Seq_2  300   TGAGTGAT-GGTTAGGATAATCAATACTCTAAATGCTGGTAGTTCTCTCTCTTGATTCAT  358

Seq_1  257   TTTTGCATCATTGCTTGTCAAAAAAGGTGGAA  288
             |||||||||||||||||||||||||||||||#
Seq_2  359   TTTTGCATCATTGCTTGTCAAAAAAGGTGGA-  389
```

Supplementary Figure 2. 102 bp sequence insertion in Cl. 14. Seq_1 refers to the gene-corrected clone SNCA Cl.33. Seq_2 refers to Cl.14. The highlighted “grey” sequence refers to the 102 bp insertion. The highlighted “blue” base pair at 126 bp refers to the site of the A30P mutation.
