# Supplementary material for: Using High-Content Screening to Generate Single-Cell Gene-Corrected Patient-Derived iPS Clones Reveals Excess Alpha-Synuclein with Familial Parkinson’s Disease Point Mutation A30P
Source: Cells. 2020 Sep 10;9(9):2065. doi: 10.3390/cells9092065 (PMC7564375; doi:10.3390/cells9092065)
Supplement: Supplementary file 1 [file cells-09-02065-s001.zip › Supp Figures/Supp. Figure 3.pdf]

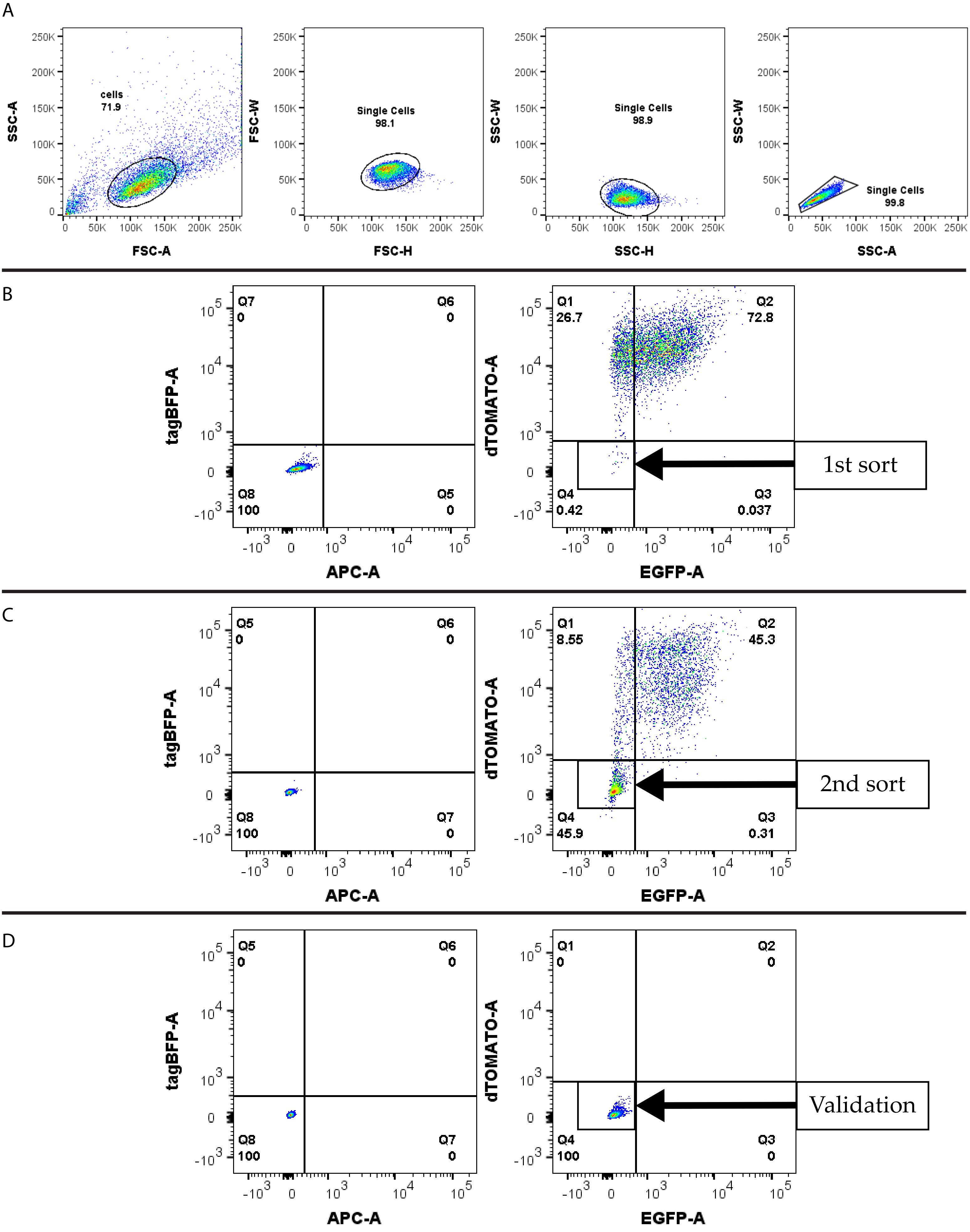

Supplementary Figure 3. Transposase removal of biallelic constructs using FACS and validation of footprint-free isogenic cell lines. (A) Set up of gating parameters for sorting a live cell population with doublet-discrimination, (B) Left panel shows tagBFP- cells, right panel shows iPSCs expressing both dTOMATO and EGFP fluorescent constructs. Gate and arrow indicate sorted cell population (0.42%) after the transposase removal of the construct. The cells sorted were expanded and resorted in (C), left panel shows no BFP cells, the right panel contains a mixed population with and without the fluorescent constructs, 45.9% of the cells sorted in (B) had the constructs been successfully excised, this population (black arrow) is then resorted and purified. (D) Right panel, 100% of the cells sorted in step C (black arrow) have had their constructs excised.
