# Supplementary material for: Using High-Content Screening to Generate Single-Cell Gene-Corrected Patient-Derived iPS Clones Reveals Excess Alpha-Synuclein with Familial Parkinson’s Disease Point Mutation A30P
Source: Cells. 2020 Sep 10;9(9):2065. doi: 10.3390/cells9092065 (PMC7564375; doi:10.3390/cells9092065)
Supplement: Supplementary file 1 [file cells-09-02065-s001.zip › Supp Figures/Supp. Figure 4_SNCA4-A30P clone 5.pdf]

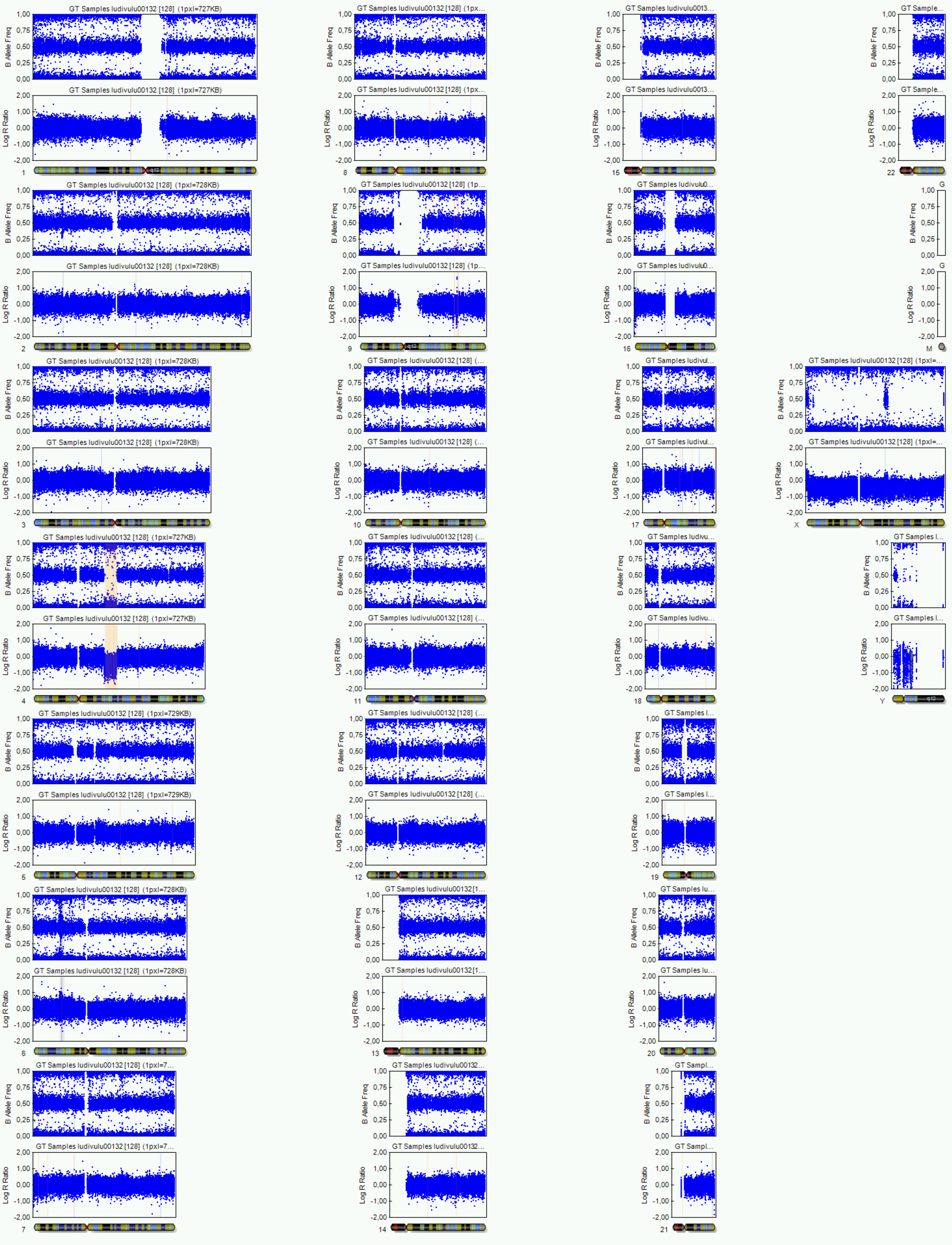

Supplementary Figure 4: Molecular karyotype of the single-cell gene-corrected cell line Clone 5. Single nucleotide polymorphism assay shows a 14Mbp deletion on the long arm of Chromosome 4.
