# Supplementary material for: Using High-Content Screening to Generate Single-Cell Gene-Corrected Patient-Derived iPS Clones Reveals Excess Alpha-Synuclein with Familial Parkinson’s Disease Point Mutation A30P
Source: Cells. 2020 Sep 10;9(9):2065. doi: 10.3390/cells9092065 (PMC7564375; doi:10.3390/cells9092065)
Supplement: Supplementary file 1 [file cells-09-02065-s001.zip › Supp Figures/Supp. Figure 8 Tuj1TH gating.pdf]

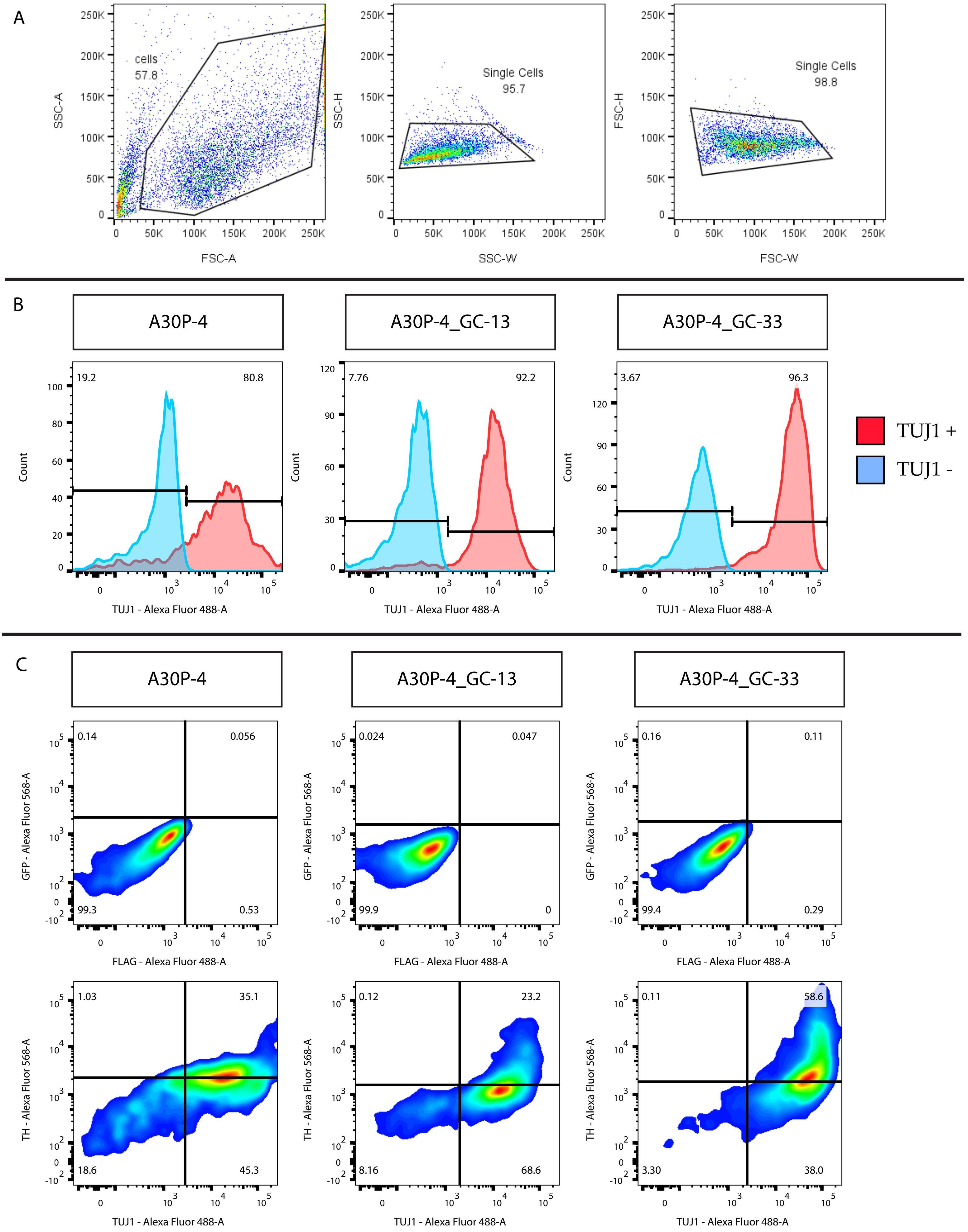

Supplementary Figure 8: Gating used to analyse neuronal expression by flow cytometry analysis. (A) Gating parameters used to isolate a single-cell population for analysis. (B) A representative histogram plot showing TUJ1 positive neurons. (C) Co-localisation analysis of the dopaminergic marker Tyrosine Hydroxylase (TH) and neuronal marker TUJ1. Antibodies against FLAG and GFP were used as negative controls.
