# Supplementary material for: Using High-Content Screening to Generate Single-Cell Gene-Corrected Patient-Derived iPS Clones Reveals Excess Alpha-Synuclein with Familial Parkinson’s Disease Point Mutation A30P
Source: Cells. 2020 Sep 10;9(9):2065. doi: 10.3390/cells9092065 (PMC7564375; doi:10.3390/cells9092065)
Supplement: Supplementary file 1 [file cells-09-02065-s001.zip › Supp Figures/Supplementary table 1_Excision efficiency table.pdf]

Supplementary Table 1: Transposons-mediated excision efficiency

Excision rate (EGFP-/DTOMATO-)				
Cell line	1st sort	2nd sort	3rd sort	4th sort
A30P-4 edited clone 5	0.24%	45.90%	100%	
A30P-4 edited clone 13	0.11%	43.60%	100%	
A30P-4 edited clone 33	0.58%	2.13%	98.40%	100%
A30P-4 edited clone 21	0.42%	12.60%	97%	100%
A30P-4 edited clone 4	0.19%	2.68%	99.50%	
Average	**0.31%**	**21.38%**	**99.98%**	**100%**
